# Does 3D-Assisted Acetabular Fracture Surgery Improve Surgical Outcome and Physical Functioning?—A Systematic Review

**DOI:** 10.3390/jpm11100966

**Published:** 2021-09-27

**Authors:** Anne M. L. Meesters, Neeltje M. Trouwborst, Jean-Paul P. M. de Vries, Joep Kraeima, Max J. H. Witjes, Job N. Doornberg, Inge H. F. Reininga, Frank F. A. IJpma, Kaj ten Duis

**Affiliations:** 1 Department of Trauma Surgery, University Medical Center Groningen, University of Groningen, 9713 GZ Groningen, The Netherlands; n.m.trouwborst@umcg.nl (N.M.T.); i.h.f.reininga@umcg.nl (I.H.F.R.); f.f.a.ijpma@umcg.nl (F.F.A.I.); k.ten.duis@umcg.nl (K.t.D.); 2 Department of Surgery, University Medical Center Groningen, University of Groningen, 9713 GZ Groningen, The Netherlands; j.p.p.m.de.vries@umcg.nl; 3 3D Lab, Department of Oral and Maxillofacial Surgery, University Medical Center Groningen, University of Groningen, 9713 GZ Groningen, The Netherlands; j.kraeima@umcg.nl (J.K.); m.j.h.witjes@umcg.nl (M.J.H.W.); 4 Department of Orthopaedic Surgery, University Medical Center Groningen, University of Groningen, 9713 GZ Groningen, The Netherlands; j.n.doornberg@umcg.nl

**Keywords:** acetabular fracture, 3D, three-dimensional, 3D print, surgical planning, systematic review

## Abstract

Three-dimensional technology is increasingly being used in acetabular fracture treatment. No systematic reviews are available about the added clinical value of 3D-assisted acetabular fracture surgery compared to conventional surgery. Therefore, this study aimed to investigate whether 3D-assisted acetabular fracture surgery compared to conventional surgery improves surgical outcomes in terms of operation time, intraoperative blood loss, intraoperative fluoroscopy usage, complications, and postoperative fracture reduction, and whether it improves physical functioning. Pubmed and Embase databases were searched for articles on 3D technologies in acetabular fracture surgery, published between 2010 and February 2021. The McMaster critical review form was used to assess the methodological quality. Differences between 3D-assisted and conventional surgery were evaluated using the weighted mean and odds ratios. Nineteen studies were included. Three-dimensional-assisted surgery resulted in significantly shorter operation times (162.5 ± 79.0 versus 296.4 ± 56.0 min), less blood loss (697.9 ± 235.7 mL versus 1097.2 ± 415.5 mL), and less fluoroscopy usage (9.3 ± 5.9 versus 22.5 ± 20.4 times). The odds ratios of complications and fracture reduction were 0.5 and 0.4 for functional outcome in favour of 3D-assisted surgery, respectively. Three-dimensional-assisted surgery reduces operation time, intraoperative blood loss, fluoroscopy usage, and complications. Evidence for the improvement of fracture reduction and functional outcomes is limited.

## 1. Introduction

Acetabular fractures are fractures involving the hip socket, which might have major impacts on the patient’s mobility, social activities, and the ability to work. These severe injuries usually occur due to high-energy trauma mechanisms (i.e., car accidents) in young patients [1]. In addition, acetabular fractures are increasingly caused by low-energy trauma mechanisms (i.e., fall at ground level) in frail elderly [1]. Adequate fracture reduction and fixation is crucial to minimise the risks on progressive posttraumatic arthritis of the hip socket and the subsequent need for revision surgery to a total hip arthroplasty [2]. Acetabular fractures are complex fractures, due to the three-dimensional (3D) geometry of the pelvis and displacement of fracture fragments in multiple directions. Insight into fracture patterns can be challenging using only two-dimensional (2D) images [3]. In the past decade, 3D technology has increasingly been used in acetabular fracture treatment. Three-dimensional printing is useful for classifying acetabular fractures and for teaching purposes [4,5,6,7]. For instance, 3D printed models may improve the quality of surgical trainees’ preoperative understanding of the spatial complexity of fractures [8]. In addition, a randomised controlled trial showed that using a 3D interactive software system for teaching acetabular fracture classification improved the classification accuracy [7]. Moreover, the use of 3D printed fracture models has improved fracture classification in comparison with 2D/3D CT images, due to enhanced tactile feedback of the complex geometry [5,6]. This may result in a shorter time needed to classify the acetabular fractures and a higher interobserver agreement as compared to the evaluation of these fractures using 2D CT images [4].

Over the past few years, the number of publications on the applications of 3D-assisted surgery in acetabular fracture treatment is rapidly increasing [9]. It encompasses a spectrum of modalities, including 3D visualisation, 3D printing, and patient-specific surgical guides or implants. Preoperative planning of the fracture reduction and pre-contouring of implants using 3D printed models has been reported in acetabular fracture surgery in case series [10,11,12,13,14,15]. For example, Hu et al. [11] created virtual 3D models of fractured acetabula based on CT images and virtually reduced the fracture fragments, in order to gain more insight into fracture patterns and treatment strategies. Moreover, the uninjured hemipelvis can be mirrored virtually and 3D printed [13,15]. This printed hemipelvis can be used as a template for the pre-contouring of implants prior to surgery [13,15]. In addition, the use of 3D printed drilling guides and patient-specific osteosynthesis plates have been described [16,17,18,19]. For instance, 3D printed drilling guides have been designed to fit temporarily on top of an implant in order to aim the drill bit and screw trajectories in the predetermined directions [19]. In addition, patient-specific implants, with or without drilling guides, have been designed based on virtual 3D models [16,17]. The application of patient-specific osteosynthesis plates provides the possibility to execute the preoperative plan and attain the predetermined osteosynthesis plate and screw positions [16]. However, comparative studies or reviews on the added clinical value of 3D-assisted acetabular fracture surgery compared to conventional surgery (i.e., defined as using only radiographs and 2D CT images in preoperative planning) are only sparingly available. Next to the surgeons’ understanding of these technologies, patients cannot be informed properly about the potential benefits of these innovations. In addition, insurance companies take evidence-based decisions on the implementation of these technologies.

Therefore, a systematic review was conducted in order to assess differences in surgical outcome and physical functioning between 3D-assisted and conventional (2D) acetabular fracture treatment. Research questions were: (1) Does 3D-assisted acetabular fracture surgery compared to conventional surgery improve surgical outcomes in terms of operation time, intraoperative blood loss, intraoperative fluoroscopy usage, complications, and postoperative fracture reduction?; and (2) does 3D-assisted acetabular fracture surgery compared to conventional surgery improve physical functioning in terms of Patient-Reported Outcome Measures?

## 2. Materials and Methods

The Preferred Reporting Items for Systematic Reviews (PRISMA) [20] were used. The review protocol has been registered in the PROSPERO International prospective register of systematic reviews under registration number CRD42021225274.

### 2.1. Search Strategy and Study Selection

On 1 March 2021, the PubMed and Embase libraries were searched for articles published between 1 January 2010 and 28 February 2021. Together with a medical librarian, the search string was generated (Table 1).

Studies that were eligible for inclusion were randomised controlled trials, cohort studies, case-control studies, cross-sectional studies, and case series on the treatment of acetabular fractures in humans by using 3D technology. Exclusion criteria were reviews; letters to the editor or conference abstracts; cadaveric studies; case reports (N < 10); paediatric studies (age < 18 years); studies in other languages than English, German, French, or Dutch; studies on fracture classification, measurements or education; studies on intraoperative imaging or surgical navigation; and biomechanical studies. Articles were imported into Rayyan QCRI, a web-based sorting tool for systematic literature reviews [21]. Next, two reviewers (AM, FIJ) independently screened the articles for eligibility based on the titles and abstracts using the Rayyan QCRI tool. The same reviewers independently screened all remaining articles by full text. Finally, the references of the included articles were screened for additional relevant manuscripts.

### 2.2. Quality Check and Data Extraction

The guidelines of the McMaster University Occupational Therapy Evidence-Based Practice Research Group were used to assess the methodological quality and risk of bias [22]. The McMaster critical appraisal consists of components considering the study purpose, background literature, study design, sample size, randomisation, outcome measures, study intervention, study results, conclusions, and implications. Scores were given with ‘yes = 1 point’, ‘no = 0 points’, and ‘not applicable (NA)’. The total score reflects the methodological quality with a maximum score of 16 for RCTs, 12 for case series, and 14 for other designs. The definitive score is presented as a percentage that varies from 0 to 100%, with a higher score indicating a higher methodological quality. Scores of <50% are considered poor-quality studies, scores of 50–74% are considered moderate-quality studies, scores of 75–90% are considered good-quality studies, and scores of >90% are considered excellent-quality studies. The data extraction and quality check were independently conducted (AM, FIJ) using the McMaster Critical Review Form. Disagreements were resolved in a consensus meeting.

### 2.3. Outcome Measures

The primary outcome of this systematic review was the surgical outcome in terms of operation time, intraoperative blood loss, intraoperative fluoroscopy usage, complications, and fracture reduction. Complications were defined as nerve injury, vascular injury, infection, thrombosis/embolism, heterotopic ossification, osteoarthritis, avascular necrosis of the femoral head, and implant failure. The quality of acetabular fracture reduction was defined by the greatest residual gap or step-off at the acetabulum on the plain radiographs or on a postoperative CT scan in any of the views [23,24]. The residual displacement was graded according to Matta’s criteria as anatomic (0 to 1 mm gap and/or step-off), imperfect (2 to 3 mm), or poor (>3 mm) [24]. An adequate reduction was defined as the Matta category anatomical and satisfactory or a postoperative displacement of ≤2 mm, and a poor reduction was defined as the Matta category poor or a postoperative displacement of >2 mm. Secondary outcome was physical functioning, assessed with the Patient-Reported Outcome Measures (PROMs) or clinician-reported outcome measures. Functional outcome was graded according to the definitions of the Modified Merle d’Aubigné (Excellent 18, Good 15–17, Fair 13–14, Poor < 13) and the Harris Hip score (Excellent 90–100, Good 80–90, Fair 70–80, Poor <70) [25,26,27,28].

### 2.4. Statistical Analysis

The weighted mean with a standard deviation of all applicable studies was calculated, using SPSS (version 23, IBM, Chicago, IL, USA), when more than two studies reported the outcome variable. For comparative studies, the differences in continuous outcome measures were calculated by using the inverse variance weighting method and presented as the weighted mean difference (WMD) with the 95% confidence interval (CI), using Review Manager (version 5.4.1, The Nordic Cochrane Centre, The Cochrane Collaboration, Copenhagen, Denmark). For dichotomous variables, the odds ratio with the 95% CI was calculated using the Mantel–Haenszel method in Review Manager. A *p*-value of <0.05 was considered to indicate statistical significance. Authors were contacted to retrieve additional data, such as not reported means or their standard deviations, but retrieving additional data was unsuccessful.

## 3. Results

### 3.1. Search and Study Characteristics

**Figure 1:**
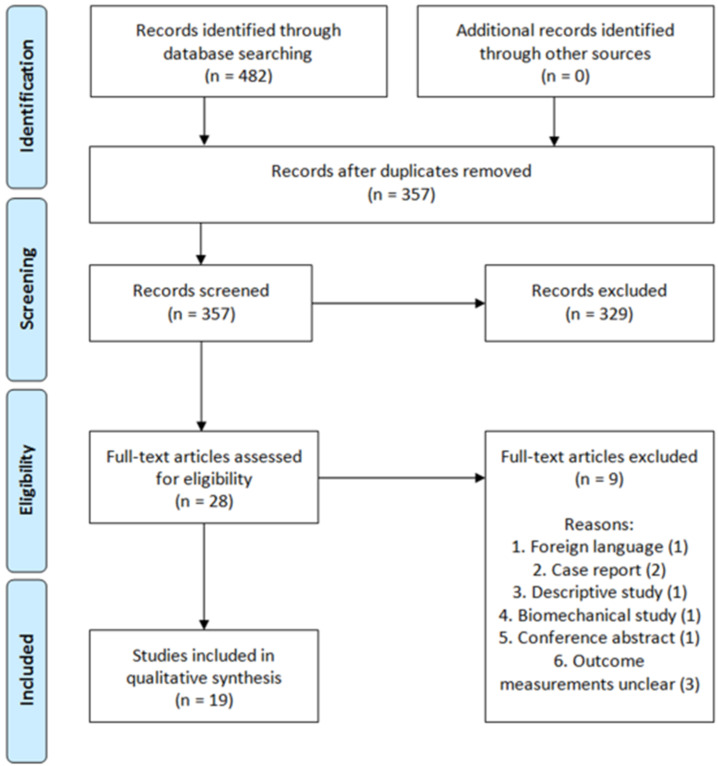
PRISMA flow diagram.

**Figure 2:**
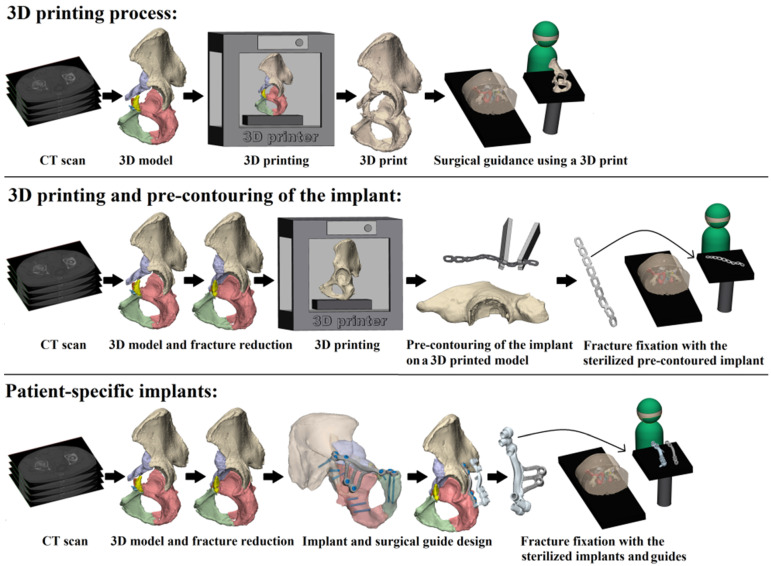
Three-dimensional-assisted surgery. Three-dimensional-assisted surgery encompasses a spectrum of modalities, including 3D visualisation, 3D printing, and patient-specific surgical guides or implants. The steps required for 3D printing, 3D printing and pre-contouring of the implant, or the manufacturing of patient-specific implants are illustrated. In the 3D printing process (top row) a virtual 3D model is created from a CT scan, e.g., using Mimics Medical software in which a threshold for bone tissue is selected based on the Hounsfield Units of the CT scan. The 3D models are split into the separate fragments, indicated by the different colours. This virtual model can be 3D printed and used for preoperative planning and surgical guidance. For 3D printing and pre-contouring of the implant (middle row), a virtual 3D model is created from a CT scan. Then, the contralateral healthy hemipelvis is mirrored, e.g., using 3-matic Medical software, and it is used as a template for the virtual fracture reduction. The fracture fragments are virtually reduced to their original anatomical position. The mirrored or virtually reduced hemipelvis can be 3D printed and this 3D print is used for pre-contouring of the implant. One study performed virtual plating and printed the contour of a plate, which was then used for pre-contouring the implant [44]. Next, the pre-contoured implant is sterilised and used for intraoperative fracture fixation. Finally, patient-specific implants (bottom row) are designed, based on the virtual 3D model from the CT scan. Either the mirrored contralateral pelvis or the fracture reduction can be used as a model for the implants. The screw directions and positions are predetermined and then the implant is designed based on the shape of the pelvis of the individual patient and based on the fracture type. The implant is accompanied by a surgical guide, to ensure that the screws are positioned and directed as planned. The implants and surgical guides are sterilised and used for intraoperative fracture fixation within four days.

In total, 482 studies were found. After removal of duplicates, 357 studies were screened on title and abstract. After title and abstract screening, 28 articles were included for full-text screening. Nine of these full-text articles were excluded due to the following reasons: foreign language article on 3D printing and pre-contouring the implant (N = 1); case reports (N = 2); descriptive study (N = 1); biomechanical study (N = 1); conference abstract (N = 1); outcome measurements unclear (N = 3). In total, 19 studies met the inclusion criteria for this systematic review (Figure 1) [29,30,31,32,33,34,35,36,37,38,39,40,41,42,43,44,45,46,47]. The included studies enrolled a total of 753 patients (median sample size 27; range 10–146). Three-dimensional-assisted surgery was used in 478 of all the patients (Figure 2). In 400 patients, a 3D print and plate pre-contouring of the implant was used (14 studies); in 69 patients, a patient-specific implant was used (three studies); and in 9 patients, only 3D printing for pre- and intraoperative fracture visualisation was used (one study). Conventional surgery, defined as preoperative planning based on radiographs and 2DCT images (axial, sagittal, and coronal views), was used in 275 patients. The study characteristics are presented in Table 2.

### 3.2. Methodological Quality Assessment

Three randomised controlled trials [38,43,44], one prospective cohort study [40], ten case control studies [30,31,32,34,35,41,42,45,46,47], and five case series [29,33,36,37] were included. The methodological quality of the papers varied from low (Table 3) to good (Table 4). The median and interquartile range (IQR) McMaster score was 69% (IQR 64–86) for all studies together and for the prospective and retrospective studies separately.

### 3.3. Surgical Outcomes

**Figure 3:**
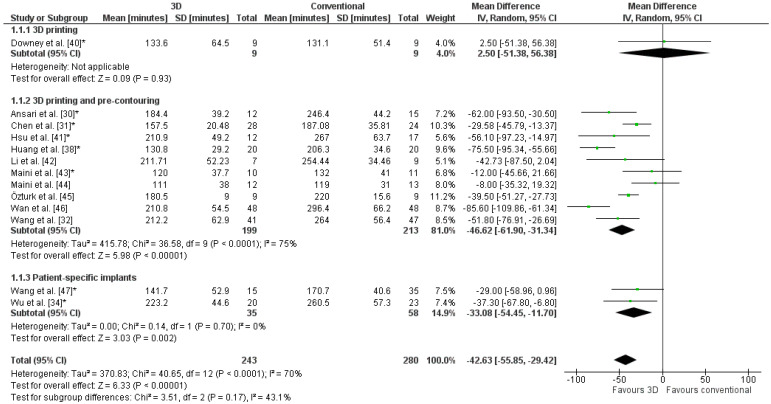
Forest plot of operation time. *: Good-quality study.

**Figure 4:**
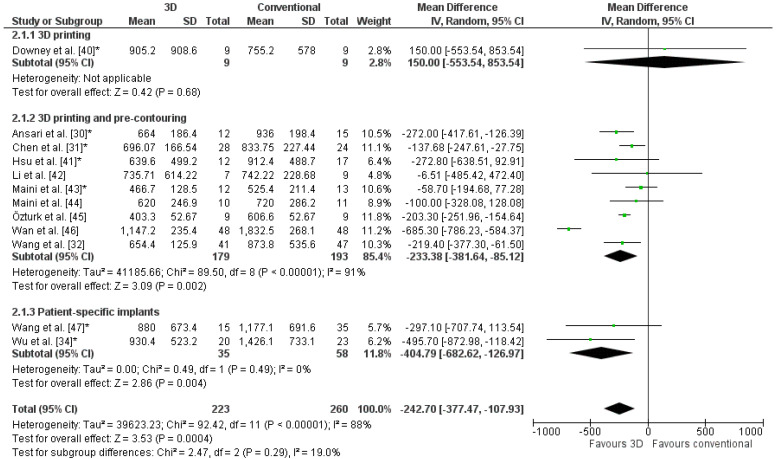
Forest plot of blood loss. *: Good-quality study.

The weighted mean operation time in the 3D-assisted group and in the conventional group was 162.5 ± 79.0 min versus 296.4 ± 56.0 min. Additionally, the weighted mean blood loss of all studies was 697.9 ± 235.7 mL versus 1097.2 ± 415.5 mL. Nine out of fourteen comparative studies reported a significantly shorter operation time and less blood loss when 3D-assisted surgery was performed [30,31,32,34,38,41,45,46,47]. The operation time was 43 min shorter for the 3D-assisted group compared to the conventional group, but the heterogeneity was high (Figure 3). There was 243 mL less blood loss in the 3D-assisted group compared to the conventional group, but the heterogeneity was high (Figure 4).

The weighted mean of three studies reporting on fluoroscopy frequency was 9.3 ± 5.9 times in the 3D-assisted group and 22.5 ± 20.4 times in the conventional group [30,45,46]. Additionally, one study using 3D prints reported a fluoroscopy dose of 1078.1 ± 800.3 mGycm^2^ in the 3D-assisted group and 727.1 ± 349.4 mGycm^2^ in the conventional group [40]. In addition, one study using 3D prints and pre-contouring of implants reported a significant decrease (*p* < 0.001) in fluoroscopy time in the 3D-assisted group (4.2 ± 1.8 s) compared to the conventional group (7.7 ± 2.6 s) [38].

**Figure 5:**
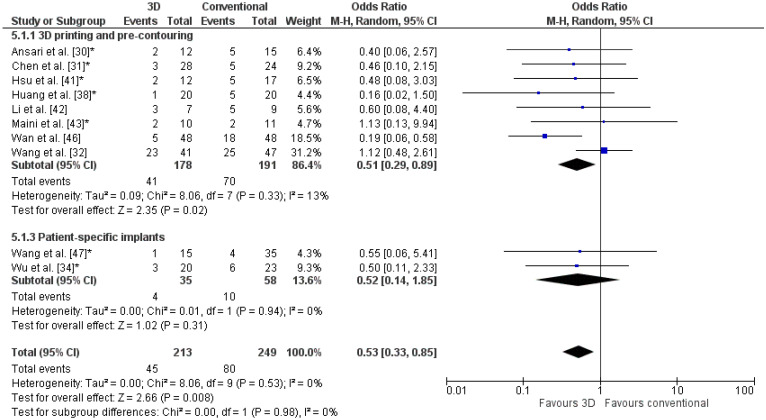
Forest plot of the complications. *: Good-quality study.

The odds ratio for complications was significantly lower for 3D-assisted surgery (OR: 0.5, Figure 5). Two studies reported no complications in both groups [40,45]. For the comparative studies using 3D printing and pre-contouring of the implants, 41 out of 187 patients (22%) had a complication in the 3D-assisted group, compared to 70 out of 200 patients (35%) in the conventional group. In the two comparative studies using patient-specific implants, four complications (11%) occurred in the 3D-assisted group, compared to ten complications (19%) in the conventional group.

**Figure 6:**
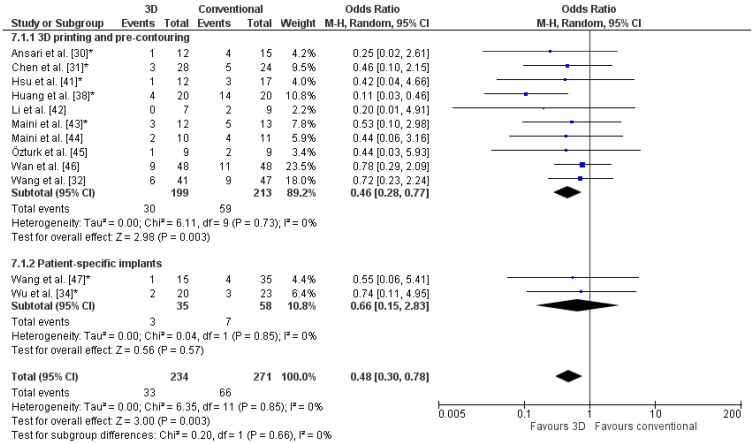
Forest plot of the postoperative reduction, where the events indicate a poor reduction. *: Good-quality study.

The weighted mean of the residual fracture displacement was 3.1 ± 1.4 (range 2–5) mm for the 3D-assisted group and 3.7 ± 2.0 (range 2–8) mm for the conventional group. The odds ratio of a poor reduction was significantly lower for 3D-assisted surgery (OR: 0.5, Figure 6). Two studies reported a better reduction in the 3D-assisted group compared to the conventional group (*p* = 0.001 and *p* = 0.003) [38,47]. In the 3D-assisted group, 14% of the patients had a poor reduction compared to 24% in the conventional group.

### 3.4. Functional Outcome

**Figure 7:**
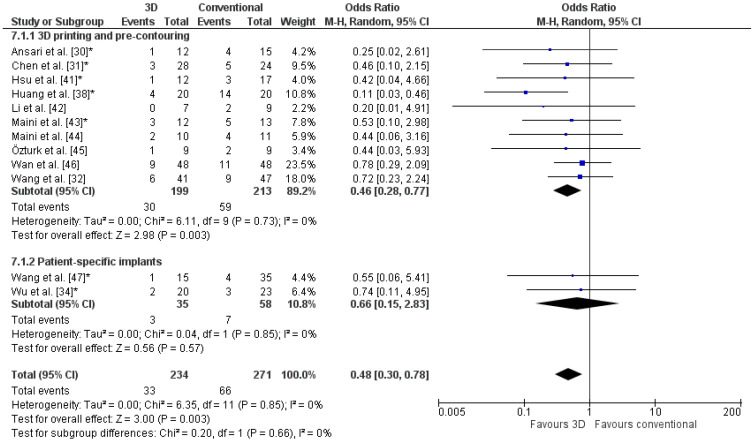
Forest plot of the functional outcome, where the events indicate a poor functional outcome. The Harris Hip score was used by Huang et al. [38] and Wan et al. [46]. The Merle d’Aubigné score was used by Wang et al. [32] and Wu et al. [34]. *: Good-quality study.

Studies that reported on functional outcome used the Harris Hip or Merle d’Aubigné scores. One study reported a Harris Hip score of 79.7 ± 13.7 in the 3D-assisted group and 83.4 ± 12.3 in the conventional group [30]. Another study reported a Modified Merle d’Aubigné score of 16.25 ± 1.64 for the 3D-assisted group and 15.83 ± 1.88 for the conventional group [31]. A significantly lower odds ratio for poor functional outcome was found for 3D-assisted surgery (OR: 0.4, Figure 7). In these studies, 84% of the patients had a good clinical outcome in the 3D-assisted group, compared to 71% in the conventional group.

## 4. Discussion

Three-dimensional-assisted surgery encompasses a spectrum of modalities, including 3D visualisation, 3D printing, and patient-specific implants, which can be implemented in the pre- and perioperative phases in acetabular fracture surgery. The added clinical value of 3D-assisted acetabular fracture surgery compared to conventional surgery is still under debate. Therefore, the aim of this systematic review was to assess whether 3D-assisted surgery improves the surgical outcome and physical functioning. Nineteen articles, using either 3D printing, 3D printing and pre-contouring of the implant, or custom-made patient-specific implants, were included in this systematic review. The results indicate a positive effect of 3D-assisted surgery on operation time, blood loss, fluoroscopy usage, and complications. Evidence of the improvement in physical functioning and fracture reduction is limited.

Shorter operation time, less intraoperative blood loss, and reduced intraoperative fluoroscopy usage in the 3D-assisted group could be explained by a more efficient surgery due to meticulous preoperative planning. Three-dimensional fracture visualisation and 3D printing give more insight into the fracture characteristics [48]. In addition, the use of 3D technology allows for the planning of screw and implant positions and to subsequently discuss it with seniors prior to the surgery. Due to optimised preparation, screw or implant malposition might be avoided. Moreover, the use of pre-contoured or patient-specific implants might contribute to efficiency as well, because time-consuming intra-operative bending and fitting manoeuvres are no longer necessary [39]. Finally, the quality of the fracture reduction is an important predictor for long-term native hip survivorship [2]. Verbeek et al. [2] found that 3% of patients with an anatomic reduction (0–1 mm of residual displacement) on CT had conversion to THA compared with 14% with an imperfect reduction (2–3 mm), and 36% with a poor reduction (>3 mm). In this review, small differences in fracture reduction were found between 3D-assisted and conventional surgery (3.1 ± 1.4 mm versus 3.7 ± 2.0 mm). Our results were difficult to compare with other large cohort studies due to differences between studies regarding imaging modalities, measurement methods, and reduction criteria [49,50]. The hypothesis was that the positive effect on fracture reduction in the 3D-assisted group could be attributed to preoperative planning of the reduction strategy and an optimal fit of the pre-contoured or patient-specific implants, which possibly serves as a reference for the fracture reduction. Larger trials are needed to assess the effect of 3D-assisted surgery on fracture reduction.

In this systematic review, six comparative studies reported on the functional outcome [30,31,32,34,38,46]. Overall, little difference in functional outcome was found after 3D-assisted versus conventional surgery (84% versus 71% good functional outcome). Some studies used the Harris Hip score [30,38,46], whereas others used the Merle d’Aubigné score [31,32,34]. Both instruments are, however, not designed and validated for evaluating the functional outcome of acetabular fractures [51]. Comparing results between studies is difficult due to the limited number of studies reporting on the functional outcome and usage of different measurement methods. Three-dimensional-assisted surgery has advantages, but it also takes effort to implement in the workflow. Additional time is needed for preoperative planning. The manufacturing of a 3D print of part of the pelvis for pre-contouring of the implant takes about six to eight hours for printing. This process is often performed and optimised by a team of technical physicians and engineers with expertise of the 3D software and hardware. Software can be either freely available online or CE-certified for medical use, with varying accompanying costs. A simple 3D printer can be used for producing in-hospital nonsterile 3D prints. However, for producing a 3D print for sterile use, one needs a medically certified 3D printer that is more expensive and often operated by an external party. Therefore, future studies about the cost-effectiveness of the 3D technologies are probably needed before they can be used on a large scale.

Limitations of this systematic review include a number of low-quality studies, small patient groups, and the heterogeneity of study populations. Moreover, possible publication bias exists because most studies solely reported positive effects of 3D-assisted surgery. However, all studies were physician-initiated and no studies were sponsored by the industry. More extensive preparation for surgery may contribute to the positive effects on the surgical and functional outcomes in addition to the 3D technology itself. A limitation of using fracture reduction as an outcome measure is that the inter- and intra-observer variabilities of the gap and step-off measurements are high [52]. Moreover, some studies assessed fracture reduction on X-rays, whereas other studies used CT scans, making it difficult to compare and interpret results and causing heterogeneity.

## 5. Conclusions

The techniques currently used in 3D-assisted acetabular fracture surgery are 3D printing and visual surgical planning, 3D printing and pre-contouring of implants, and custom-made patient-specific implants. Three-dimensional-assisted surgery compared to conventional surgery reduces operation time, intraoperative blood loss, intraoperative fluoroscopy usage, and complication rate. Evidence for the improvement of postoperative fracture reduction and physical functioning is limited, because of heterogeneity and varying qualities of the studies.

## Figures and Tables

**Table 1:** Search string.

Database	Search String
PubMed	(3D[tiab] OR three dimension*[tiab] OR 3 dimension*[tiab] OR ‘Printing, Three-Dimensional’ [Mesh] OR ‘Imaging, Three-Dimensional’ [Mesh]) AND (acetabul*[tiab] OR ‘Acetabulum’ [Mesh]) AND (fractur*[tiab] OR ‘Fractures, Bone’ [Mesh]) AND ‘2010/01/01’ [PDat]: ‘3000/12/31’ [PDat]
Embase	(‘three dimensional imaging’/exp OR ‘three dimensional printing’/exp OR ‘3 d’:ti,ab OR ‘3 dimension*’:ti,ab OR ‘three dimension*’:ti,ab) AND (‘acetabulum’/exp OR acetabul*:ti,ab) AND (‘fracture’/exp OR fractur*:ti,ab) AND [embase]/lim AND [2010,2011,2012,2013,2014,2015,2016,2017,2018,2019,2020,2021]/py

**Table 2:** Study characteristics.

Study	Year	Country	Design	N	Period	Outcome Measurements	3D Technology
Ansari et al. [30]	2020	India	Case control	27	August 2017–July 2018	Operation time, intraoperative blood loss, intraoperative fluoroscopy usage, postoperative fracture reduction, complications, FU: Harris hip score	3D printing and plate pre-contouring
Chen et al. [31]	2019	China	Case control	52	January 2013–January 2017	Operation time, intraoperative blood loss, postoperative fracture reduction, complications, FU: modified Merle d’Aubigné	3D printing and plate pre-contouring; virtual plating
Downey et al. [40]	2020	Ireland	Prospective cohort	18	October 2017–May 2018	Operation time, intraoperative blood loss, intraoperative fluoroscopy usage, postoperative fracture reduction, complications: infection	3D printing
Hsu et al. [41]	2019	China	Case control	29	March 2014–February 2018	Operation time, intraoperative blood loss, postoperative fracture reduction, complications	3D printing and plate pre-contouring
Huang et al. [38]	2020	China	Randomised Controlled Trial	40	September 2013–September 2017	Operation time, intraoperative blood loss, intraoperative fluoroscopy usage, postoperative fracture reduction, complications, FU: Harris hip score	3D printing and plate pre-contouring
IJpma et al. [39]	2021	Netherlands	Prospective case series	10	January 2017–December 2018	Postoperative fracture reduction, complications, FU: Short Musculoskeletal Function Assessment	Patient-specific implants
Li et al. [42]	2019	Taiwan	Case control	16	September 2013–August 2017	Operation time, intraoperative blood loss, postoperative fracture reduction, complications	3D printing and plate pre-contouring
Maini et al. [43]	2018	India	Randomised Controlled Trial	21	June 2012–December 2014	Operation time, intraoperative blood loss, postoperative fracture reduction, complications	3D printing and plate pre-contouring
Maini et al. [44]	2018	India	Randomised Controlled Trial	25	October 2014–March 2016	Operation time, intraoperative blood loss, postoperative fracture reduction	3D printing of virtually pre-contoured plates as template for plate pre-contouring
Öztürk et al. [45]	2020	Turkey	Case control	18	January 2017–June 2018	Operation time, intraoperative blood loss, intraoperative fluoroscopy usage, postoperative fracture reduction, complications	3D printing and plate pre-contouring
Wan et al. [46]	2019	China	Case control	96	January 2016–June 2017	Operation time, intraoperative blood loss, intraoperative fluoroscopy usage, postoperative fracture reduction, complications, FU: Harris hip score	3D printing and plate pre-contouring
Wang et al. [47]	2020	China	Case control	50	January 2016–June 2017	Operation time, intraoperative blood loss, postoperative fracture reduction, complications	Patient-specific implants
Wang et al. [32]	2020	China	Case control	88	February 2013–February 2016	Operation time, intraoperative blood loss, postoperative fracture reduction, complications, FU: Merle d’Aubigne	3D printing and plate pre-contouring
Weidert et al. [33]	2020	Germany	Retrospective case series	12	NS	Operation time, intraoperative blood loss, FU: (modified) Harris hip score, Merle d’Aubigne	3D printing and plate pre-contouring
Wu et al. [34]	2020	China	Case control	43	May 2014–January 2018	Operation time, intraoperative blood loss, postoperative fracture reduction, complications, FU: modified Merle d’Aubigne	Patient-specific implants
Xu et al. [29]	2014	China	Prospective case series	24	January 2008–August 2011	Operation time, intraoperative blood loss, postoperative fracture reduction, FU: Merle d’Aubigne, complications	Patient-specific implants
Yu et al. [35]	2020	China	Case control	146	June 2011–December 2017	Operation time, intraoperative blood loss, intraoperative fluoroscopy usage, postoperative fracture reduction, complications, FU: Harris hip score	3D printing and plate pre-contouring
Zeng et al. [36]	2016	China	Prospective case series	10	June 2013–February 2015	Postoperative fracture reduction, complications	3D printing and plate pre-contouring
Zou et al. [37]	2020	China	Retrospective case series	33	June 2017–December 2018	Operation time, intraoperative blood loss, postoperative fracture reduction, complications, FU: modified Merle d’Aubigne	3D printing and plate pre-contouring

NS = Not Addressed, FU: Follow-up methods.

**Table 3:** Quality assessment part one.

Categories	Zou, 2020	Weidert, 2020	Zeng, 2016	Öztürk, 2020	Wan, 2019	Xu, 2014	Maini, 2018 ^1^	Li, 2019	Wang, 2020 ^2^
1. Study purpose									
Was the study question clearly stated?	0	0	0	1	0	1	1	1	0
2. Literature review									
Was relevant background literature reviewed?	0	0	1	1	0	0	1	1	1
3. Study design	CR	CR	CR	CC	CC	CR	RCT	CC	CC
4. Sample									
Was the sample described in detail?	1	0	1	1	1	1	1	1	1
Was the sample justified?	0	0	0	0	0	0	0	0	0
Were the groups randomised?	0	0	0	0	0	0	1	0	0
Was randomising appropriate done?	NA	NA	NA	NA	NA	NA	1	NA	NA
5. Outcomes									
Were the outcome measures reliable?	0	1	0	0	1	1	1	0	1
Were the outcome measures valid?	0	1	1	0	1	1	1	0	1
6. Intervention									
Intervention was described in detail?	1	1	1	1	0	1	1	1	1
Contamination was avoided?	NA	NA	NA	1	1	1	1	1	1
Cointervention was avoided?	NA	NA	NA	1	1	0	1	1	1
7. Results									
Results were reported in terms of statistical significance?	0	0	0	1	1	0	0	1	1
Were the analysis method/s appropriate?	0	0	0	0	1	0	0	1	1
Clinical importance was reported?	1	1	1	1	1	1	1	1	1
Drop-outs were reported?	0	0	0	0	0	0	0	0	0
8. Conclusion									
Conclusions were appropriate given study methods and results?	0	1	0	0	0	1	0	1	0
Total	3/12	5/12	5/12	8/14	8/14	7/12	11/16	10/14	10/14
%	25	42	42	57	57	58	69	71	71

Yes = 1 point, no = 0 points, CC = Case Control study, RCT = Randomised Controlled Trial, CR = Case Series, N/A = Not applicable. ^1^: Maini et al. (2018)—Evaluation of accuracy of virtual surgical planning for patient-specific pre-contoured plate in acetabular fracture fixation. ^2^: Wang et al. (2020)—The effect of new preoperative preparation method compared to conventional method in complex acetabular fractures: minimum 2-year follow-up.

**Table 4:** Quality assessment part two.

Categories	Huang, 2020	Maini, 2018 ^3^	Wu, 2020	IJpma, 2021	Ansari, 2020	Chen, 2019	Downey, 2020	Hsu, 2019	Wang, 2020 ^4^	Yu, 2020
1. Study purpose										
Was the study question clearly stated?	0	1	1	1	1	1	1	1	1	1
2. Literature review										
Was relevant background literature reviewed?	1	1	0	1	1	1	1	1	1	0
3. Study design	RCT	RCT	CC	CS	CC	CC	CS	CC	CC	CC
4. Sample										
Was the sample described in detail?	1	1	1	1	1	1	1	1	1	1
Was the sample justified?	0	0	0	0	0	0	0	0	0	0
Were the groups randomised?	1	1	0	0	0	0	0	0	0	0
Was randomising appropriate done?	0	1	NA	NA	NA	NA	NA	NA	NA	NA
5. Outcomes										
Were the outcome measures reliable?	1	1	1	1	1	1	1	1	1	1
Were the outcome measures valid?	1	0	1	1	1	1	1	1	1	1
6. Intervention										
Intervention was described in detail?	1	1	1	1	1	1	1	1	1	1
Contamination was avoided?	1	1	1	NA	1	1	1	1	1	1
Cointervention was avoided?	1	1	1	NA	1	1	1	1	1	1
7. Results										
Results were reported in terms of statistical significance?	1	1	1	1	1	1	1	1	1	1
Were the analysis method/s appropriate?	1	1	1	1	1	1	1	1	1	1
Clinical importance was reported?	1	1	1	1	1	1	1	1	1	1
Drop-outs were reported?	0	0	0	0	0	0	0	0	0	1
8. Conclusion										
Conclusions were appropriate given study methods and results?	1	0	1	1	1	1	1	1	1	1
Total	12/16	12/16	11/14	10/12	12/14	12/14	12/14	12/14	12/14	12/14
%	75	75	79	83	86	86	86	86	86	86

Yes = 1 point, no = 0 points, CC = Case Control study, CS = Cohort study, RCT = Randomised Controlled Trial, CR = Case Series, N/A = Not applicable. ^3^: Maini et al. (2018)—Three-dimensional printing and patient-specific pre-contoured plate: future of acetabulum fracture fixation? ^4^: Wang et al. (2020)—Three-dimensional printing of patient-specific plates for the treatment of acetabular fractures involving quadrilateral plate disruption.

## Data Availability

The authors declare that the data supporting the findings of this study are available within the paper.
